# Interfacial Adsorption Kinetics of Methane in Microporous Kerogen

**DOI:** 10.1021/acs.langmuir.2c03485

**Published:** 2023-03-01

**Authors:** Runxi Wang, Saikat Datta, Jun Li, Saad F. K. Al-Afnan, Livio Gibelli, Matthew K. Borg

**Affiliations:** † Institute of New Energy and Low-Carbon Technology, Sichuan University, Chengdu 610065, China; ‡ School of Engineering, Institute of Multiscale Thermofluids, The University of Edinburgh, Edinburgh EH9 3FB, United Kingdom; ¶ Center for Integrative Petroleum Research, College of Petroleum Engineering and Geosciences, King Fahd University of Petroleum and Minerals, Dhahran 31261, Saudi Arabia

## Abstract

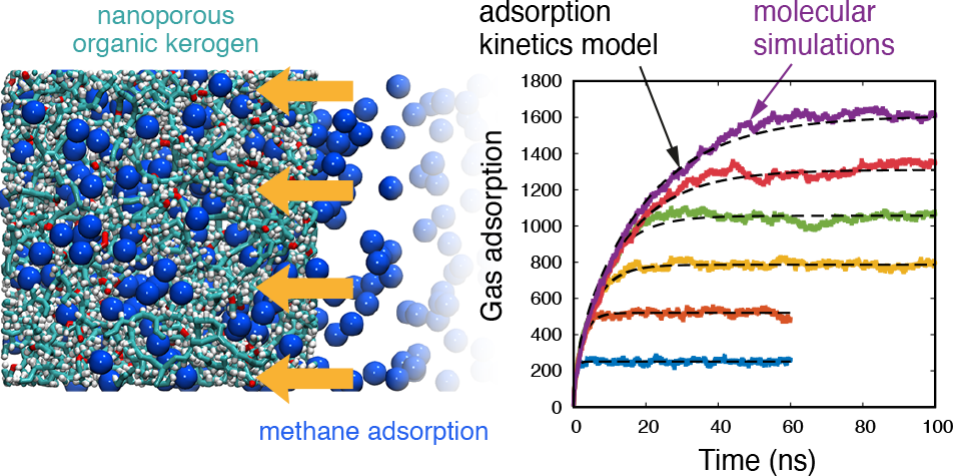

Rapid declines in unconventional shale production arise from the poorly understood interplay between gas transport and adsorption processes in microporous organic rock. Here, we use high-fidelity molecular dynamics (MD) simulations to resolve the time-varying adsorption of methane gas in realistic organic rock samples, known as kerogen. The kerogen samples derive from various geological shale fields with porosities ranging between 20% and 50%. We propose a kinetics sorption model based on a generalized solution of diffusive transport inside a nanopore to describe the adsorption kinetics in kerogen, which gives excellent fits with all our MD results, and we demonstrate it scales with the square of the length of kerogen. The MD adsorption time constants for all samples are compared with a simplified theoretical model, which we derive from the Langmuir isotherm for adsorption capacitance and the free-volume theory for steady, highly confined bulk transport. While the agreement with the MD results is qualitatively very good, it reveals that, in the limit of low porosity, the diffusive transport term dominates the characteristic time scale of adsorption, while the adsorption capacitance becomes important for higher pressures. This work provides the first data set for adsorption kinetics of methane in kerogen, a validated model to accurately describe this process, and a qualitative model that links adsorption capacitance and transport with the adsorption kinetics. Furthermore, this work paves the way to upscale interfacial adsorption processes to the next scale of gas transport simulations in mesopores and macropores of shale reservoirs.

## Introduction

Compared with conventional gas fields, shale gas extraction is still not well predicted and understood.^1^ Natural production of methane gas occurs through chemical-reaction processes inside the sedimentary rock called kerogen, which adsorbs inside the nanopores^2^ owing to the intermolecular van der Waals forces. Adsorption is well-known to depend on the formation pressure, temperature, and the properties of the organic matter.^3−6^ In production, hydrofracking is often used to improve the connectivity of the ultralow permeability pores inside the kerogen matrix, which increases the mobility of the gas and the gas recovery.^7^ Despite turning to these intense stimulating techniques, the productivity of shale reservoirs has often been found to rapidly reduce with time during gas production, making forecasting of a reservoir unmanageable and unreliable.^8^ Understanding the kinetics sorption processes of methane at the interface of kerogen matter, i.e., the time-varying adsorption and desorption processes when subject to changes in local reservoir pressures, is foundational to solving the decline in extraction during shale gas production.

Experiments remain a useful part of understanding steady-state adsorption in nanopores and macropores of shale,^9^ including the impact of local porosity,^10^ the measurement of diffusion coefficients,^11^ and the impact of temperature,^12^ to name a few. However, experiments still have limitations in clearly revealing the physics of the local, dynamic transport, and adsorption processes that occur at a molecular level and are free of heterogeneous effects. Furthermore, compared with a large number of adsorption models in the literature,^9,13−15^ theoretical frameworks accurately describing the time-varying transfer of gas at an interface of a porous rock are still lacking.

Molecular dynamics (MD) simulation has unique advantages in that it can be used to study the gas transport inside nanopores with high spatial and temporal resolution. For example, using MD, the self-diffusion of hydrocarbons of varying chain length was directly measured in equilibrium simulations.^16,17^ Due to the extremely low permeability of kerogen, the transport of these hydrocarbon chains within the nanopores is found to be driven by self-diffusion, and importantly, Darcy’s law is found to no longer be valid.^9,18−21^ As the velocity cross-correlations of alkane molecules are no longer significant, the transport mechanisms are dominated by properties of the matrix and adsorbed fluid, which is in contrast to previous classical understanding, where viscosity and porosity dominate porous media flows.^16,22^ Other similar MD simulations were used to study methane displacement in carbon nanochannels,^23^ oil migration in nanopores, and methane adsorption/desorption from carbon nanotubes^24^ and kerogen.^25−27^

In this work, we use nonequilibrium molecular dynamics (NEMD) simulation to accurately measure the time-varying adsorption of methane gas molecules inside realistic organic rock samples. We derive a sorption kinetics model that best describes this adsorption process and demonstrate it fits all our MD results well. From these fits, we are able to rationalize the adsorption process using the adsorption time constant for various sample maturities, porosities, and reservoir pressures. We also use MD to validate the scaling law of the adsorption time constant, which relates to the square of the kerogen length, that arises from the sorption kinetics model. We believe the sorption kinetics model and the MD data generated for these samples will be useful when upscaling in meso/macro flow simulations,^28−31^ where boundary models of the sorption kinetics processes are currently missing. Finally, a simplified theoretical model for the adsorption time constant is developed, which we find linearly depends on the flow resistance and adsorption capacitance. This allows us to untangle the flow transport from the pore adsorption capacitance to understand its individual impact on the adsorption time constant for the parametric space considered.

## Materials and Methods

### Molecular Dynamics Simulations

**Figure 1:**
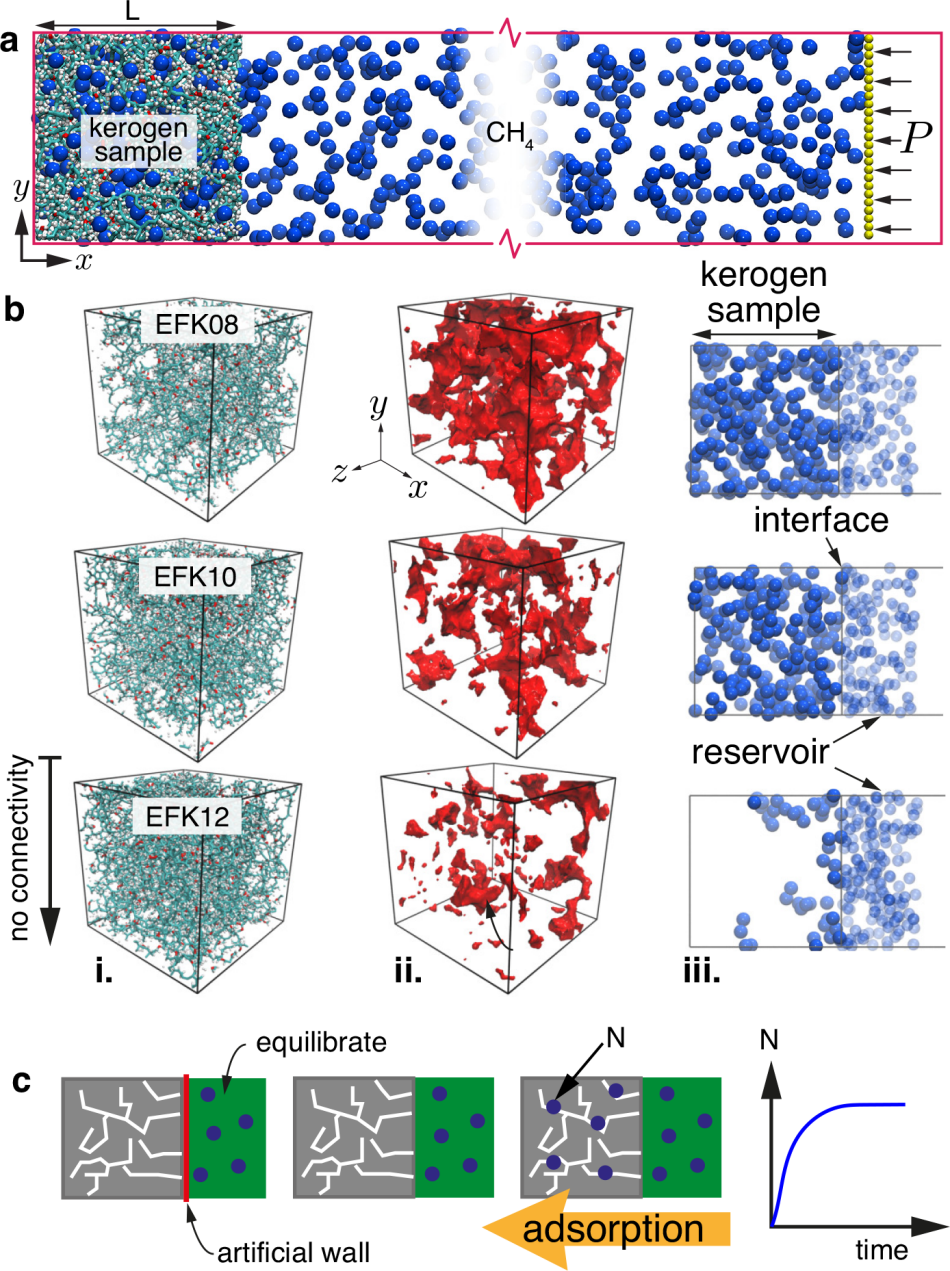
(a) Schematic of the NEMD setup for the adsorption simulations used in this work. Kerogen of length *L* equal to the sample length *L*_s_ = 50 Å shown on the left; methane (CH_4_) molecules are colored blue, and the piston on the right is colored yellow, which sets the reservoir pressure *P*. (b) (i) EFK kerogen samples at three different densities of 0.8 g/cm^3^ (EFK08), 1.0 g/cm^3^ (EFK10), and 1.2 g/cm^3^ (EFK12), (ii) their respective pore space, and (iii) side view of steady-state methane molecule adsorption in the kerogen samples for a reservoir pressure of 10 MPa; note that the kerogen is removed in (ii) and (iii) for better visualization. (c) Procedure for initializing adsorption MD simulations. Here, the kerogen sample is drawn in gray; the temporary barrier used during reservoir gas equilibration is in red, and the reservoir is highlighted green; blue circles are the methane molecules.

The time-varying adsorption of methane inside kerogen samples, which interface with a reservoir of given pressure *P*, is investigated using the NEMD setup in Figure 1a. The system is periodic in the *y* and *z* directions and nonperiodic in the *x* direction. A kerogen sample is fixed on one end of the simulation domain, and a piston is used on the other end of the domain to maintain a reservoir of methane molecules at a steady pressure, such that the direction of adsorption is in the negative *x* direction. The force applied on each piston atom is *F* = *PA*/*N*, where *P* is the target reservoir pressure varying between 1 and 50 MPa, *A* is the piston cross-sectional area, and *N* = 625 is the number of piston atoms. The direction of the piston force always points into the reservoir, while the mass of the atoms on the piston are taken to be those of carbon.

State-of-the-art molecular structures of kerogen (sample size: *L*_s_(3) = 50 × 50 × 50 Å^3^) were obtained from Bousige et al.,^32^ which derive from the Middle East (MEK, Type II), Eagle Ford field (EFK, Type II), and the Marcellus field (MarK, Type IV) reservoirs. The densities vary between 0.8 and 1.4 g/cm^3^, which translate to porosities (ϕ = 20–60%).^17^ These kerogen samples are constructed by Bousige et al.^32^ using the hybrid reverse Monte Carlo reconstruction method, which match the C–C pair distribution functions, pore size distribution, elastic properties, adsorption, and vibrational density of state with experiments. We find that samples with densities higher than ∼1.0 g/cm^3^ have no bulk connectivity (see Figure 1b for EFK12), and the adsorption will only be superficial at the interface. Therefore, in this work, we focus only on samples that have bulk connectivity (i.e., 0.8 and 1.0 g/cm^3^ densities). The force fields between kerogen and methane are obtained from experimental calibrations of adsorption of the same kerogen samples,^17,32^ where methane is modeled as a single site Lennard-Jones molecule^33^ (see Table 1 for all pair-potential combinations). Simulations are performed in the NVT ensemble using the MD software LAMMPS.^34^ The temperature of methane in all simulations is maintained at 423 K (relevant to shale reservoirs at burial depth of ∼5 km) by the Nosé–Hoover thermostat^35^ applied only in the nonflow (*y* and *z*) directions.

**Table 1:** Lennard-Jones Pair-Interaction Parameters for Energy ϵ_*ij*_ and Length Scale σ_*ij*_ between Methane (CH_4_) and Other Molecular Species: Kerogen (C, O, H) and Piston (P)^a^

molecule pairs	ϵ_*ij*_ (kcal/mol)	σ_*ij*_ (Å)
CH_4_–CH_4_	0.2941	3.730
CH_4_–C	0.12787	3.545
CH_4_–O	0.21351	3.45
CH_4_–H	0.09362	3.075
CH_4_–P	0.005	2.5
C–C	0.0556	3.360
O–O	0.1550	3.170
H–H	0.0298	2.420

a A potential cut-off of *r*_cut_ = 20 Å is used, except for the pair-interaction kerogen atoms, which use an artificially small *r*_cut_ = 0.1 Å, since their thermal motions are largely dictated by the Langevin thermostat.

**Figure 2:**
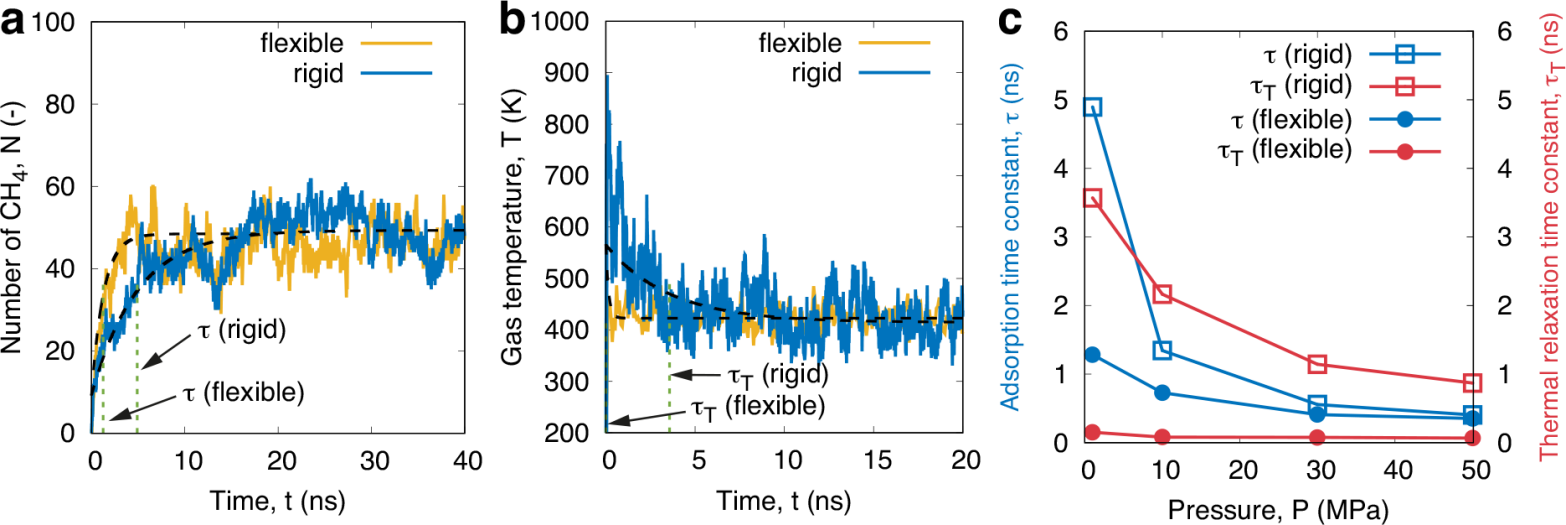
Differences in the (a) adsorption and (b) temperature of methane inside the EFK 0.8 g/cm^3^ sample at 1 MPa between rigid and flexible kerogen matrix models. (c) Comparison of the adsorption time constant (τ; blue) with the thermal relaxation time constant (τ_T_; red).

Kerogen structures have been treated as either *rigid* or *flexible* to compare their influence on the adsorption kinetics. The atoms of a rigid kerogen are fixed at their equilibrium position, while those of a flexible kerogen are modeled with a spring force (spring constant of 100 kcal/(mol Å^2^)) tethering them to their equilibrium position, and each atom is connected to a Langevin thermostat set at the same target temperature of the reservoir. The simulations of rigid kerogen are conducted with a time step of 2 fs, while the flexible model uses a smaller time step of 0.5 fs. As we detail in the Supporting Information (SI), we have chosen the flexible kerogen structure in our study, because the rigid kerogen was found to create longer adsorption time scales τ (see Figure 2). In previous work,^36,37^ differences in gas self-diffusion between flexible and rigid kerogen models were observed to originate from a combination of swelling and tight flexible constrictions that open/close within the atomic matrix. In our case, our setup does not handle swelling, and we find that an adiabatic treatment of the rigid kerogen matrix prevents the changes in heat formation in the adsorbing gas to rapidly thermalize within the kerogen, which lengthens the time of adsorption. This is resolved using a flexible and thermally constrained matrix. Figure 2 shows a comparison of the adsorption time scale τ with the corresponding thermal relaxation time scale τ_T_ of the gas during adsorption for both rigid and flexible treatments. For the rigid kerogen matrix, the thermal relaxation time scale is comparable to the adsorption time scale, while for the flexible matrix, the thermal relaxation time scale is much lower. Further details are given in the SI.

Since the kerogen structures are constructed in a periodic domain, we are also able to place multiple equivalent samples side-by-side to construct a thicker kerogen, given by *L* = *iL*_s_, where *L*_s_ = 50 Å is the original sample size, and *i* = 1, 2, ... is an integer representing the number of unit replicas.

The MD procedure is shown schematically in Figure 1c. In the initialization step, the number of methane molecules set in the reservoir are generated at the target density and pressure, taken from NIST.^38^ The equation of state has also been checked with independent MD simulations of methane as well as with the action of the piston. The size of the reservoir between piston and kerogen is chosen such that the number of methane molecules that remain in the reservoir after steady-state adsorption remains >95%. Starting from an empty kerogen, the initialized methane molecules in the reservoir are equilibrated at the target pressure and temperature for 3 ns, while the kerogen entrance is kept closed using a sheet of atoms at the entrance with an artificial weak LJ potential between the sheet and methane. The entrance to the kerogen is then opened by removing the artificial sheet, and methane is allowed to adsorb into the kerogen sample under a constant pressure for tens to hundreds of nanoseconds (depending on the properties of the gas and sample) until adsorption reaches a steady-state. Data for the variation in number of molecules as a function of time, *N*(*t*) from the start of the main simulation, as well as the temperature of gas molecules inside the kerogen *T*(*t*) are measured.

### Interfacial Sorption Kinetics Model

Recent works^16,39^ have shown that the steady transport process of hydrocarbons and carbon dioxide within nanopores is diffusion-based. From this finding, here we derive a model that best describes our setup in Figure 1a by solving the one-dimensional diffusion equation in a single straight nanopore^40^ and extend this to a general porous network, which is given by
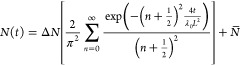
1where  is the change in the number of adsorbed gas molecules at time *t* = 0 (given by *N*_0_) and the steady-state (given by , *t* → ∞) and *L* is the length of the kerogen sample in the direction of adsorption. Note *N*_0_ = 0 in all the adsorption cases we present in the manuscript. See the SI for the full derivation.

The time constant τ of this adsorption process, and the ensuing scaling law, is given by an *L*^2^ dependence:
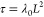
2where λ_0_ represents the intrinsic properties of the kerogen/methane characteristics, which means it depends on reservoir pressure, temperature, and the matrix properties.

**Figure 3:**
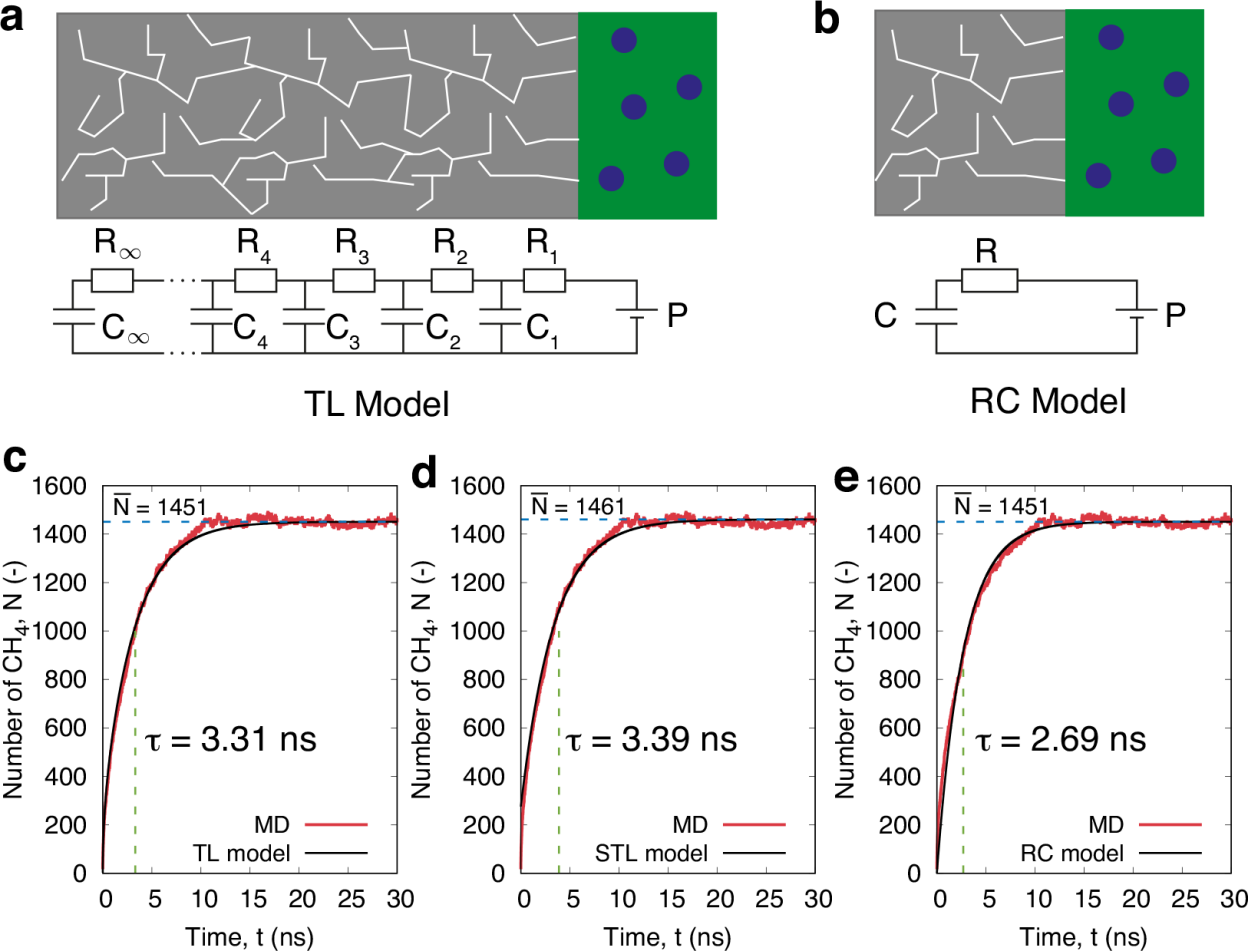
Schematic of the flow-equivalent electrical circuitry for (a) the TL model and (b) RC model used to describe the sorption gas kinetics in this work. MD fitting using the three kinetics models: (c) the TL model (eq 1), (d) the STL model (eq 3), and (e) the RC model (eq 4). The case chosen here is EFK of 0.8 g/cm^3^, *P* = 50 MPa, and kerogen length set to three times the original sample, *L* = 3 × *L*_s_, where *L*_s_ = 50 Å is the sample size. Horizontal and vertical dashed lines indicate the fitted value for  and τ, respectively, using each model. Note, τ represents ∼63% of the steady-state adsorption value, where steady-state is normally achieved at .

Equation 1 is a solution of a diffusion-type equation^41^ and is general enough to deal with heterogeneity, meaning we can apply it to porous rock setups, such as ours. The expression also has analogy with the circuit of the transmission line (TL) model in electricity, which means that we can contrast the adsorption/desorption processes of methane molecules in kerogen with the charging/discharging of electrons in an electrode. As illustrated in Figure 3a, an infinite network of resistors and capacitors connected in this way can be used to describe this process, where the resistor represents the local resistance to methane flow and the capacitor is the differential capacity of local adsorption. For ease of reference, we will refer to eq 1 as the TL model. By setting *n* = 100 terms arbitrarily, we find the TL model fits our MD data well, as we show for a random case in Figure 3c.

### Theoretical Model for τ

The TL model presented in the previous section in combination with the MD data we present in the results section is found to be a sufficient way of accounting for gas transport at boundary interfaces of kerogen with the larger reservoir pores. However, to independently verify our MD results, as well as provide a way of guiding future experimental measurements, in this section, we propose a simplified theoretical model to estimate the adsorption time constant τ.

By inspecting eq 1, we find that addends decrease exponentially with increasing *n*, and the first exponential governs the major sorption characteristics in our cases. Therefore, it is a reasonable assumption to keep just one term in eq 1 (i.e., setting *n* = 0) to obtain what we call the *simplified transmission line* (STL) model:
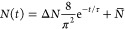
3

A representative comparison between the TL and STL models is shown in Figure 3c,d, where the time constants τ are within 3% of each other, the final steady-state adsorption value of  is within 1%, and disagreements in the STL fit arise only over a very short time interval at the beginning of the simulation because of the omitted terms in the series. We have not observed any visible differences between the TL and STL models across our data set.

Inspired from the electrical circuits origin of the TL and STL models, we find that the first-order RC model,^42,43^ which contains a resistor *R* and a capacitor *C* connected in series, as shown schematically in Figure 3b, could also satisfactorily describe the sorption kinetics in simple systems:
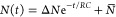
4where *R* is the flow resistance and *C* is the adsorption capacitance.

Given that the form of eq 4 looks very similar to the STL model (which we know is as accurate as the TL model), we can therefore make a calculated jump to equate the TL/STL sorption time constant with the RC model:
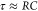
5

Note, we find that the RC model (eqs 4 and 5) produces a fit with the MD data that is visually less good, and errors are more pronounced in τ with ∼20% when compared to the TL model, as shown in Figure 3e. We find that this 20% error is a systematic error as it remains in all samples and in the upscaling study.

The resistance and capacitor terms in eq 5 are given by (see full derivation in the SI):
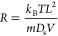
6and
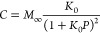
7respectively, where *k*_B_ is the Boltzmann constant, *m* is the mass of one gas molecule, *D*_s_ is the gas self-diffusion inside the kerogen, *V* is the box volume of the kerogen sample, *M*_∞_ is the saturated mass of adsorbed gas molecules, and *K*_0_ is the equilibrium constant (in units of inverse pressure). For the resistance term, we rely on a recent study^17^ that generated *D*_s_ using equilibrium MD simulations for the same kerogen samples within the free-volume framework.^16^ The origin of the capacitance term arises from the derivative of the Langmuir adsorption isotherm with respect to pressure. The derivations of both *R* and *C* terms are given in the SI.

Comparison with the scaling law (2), gives a theoretical expression for λ_0_:
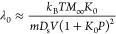
8

## Results and Discussion

### Validity of the Adsorption Kinetics Model for Upscaling

**Figure 4:**
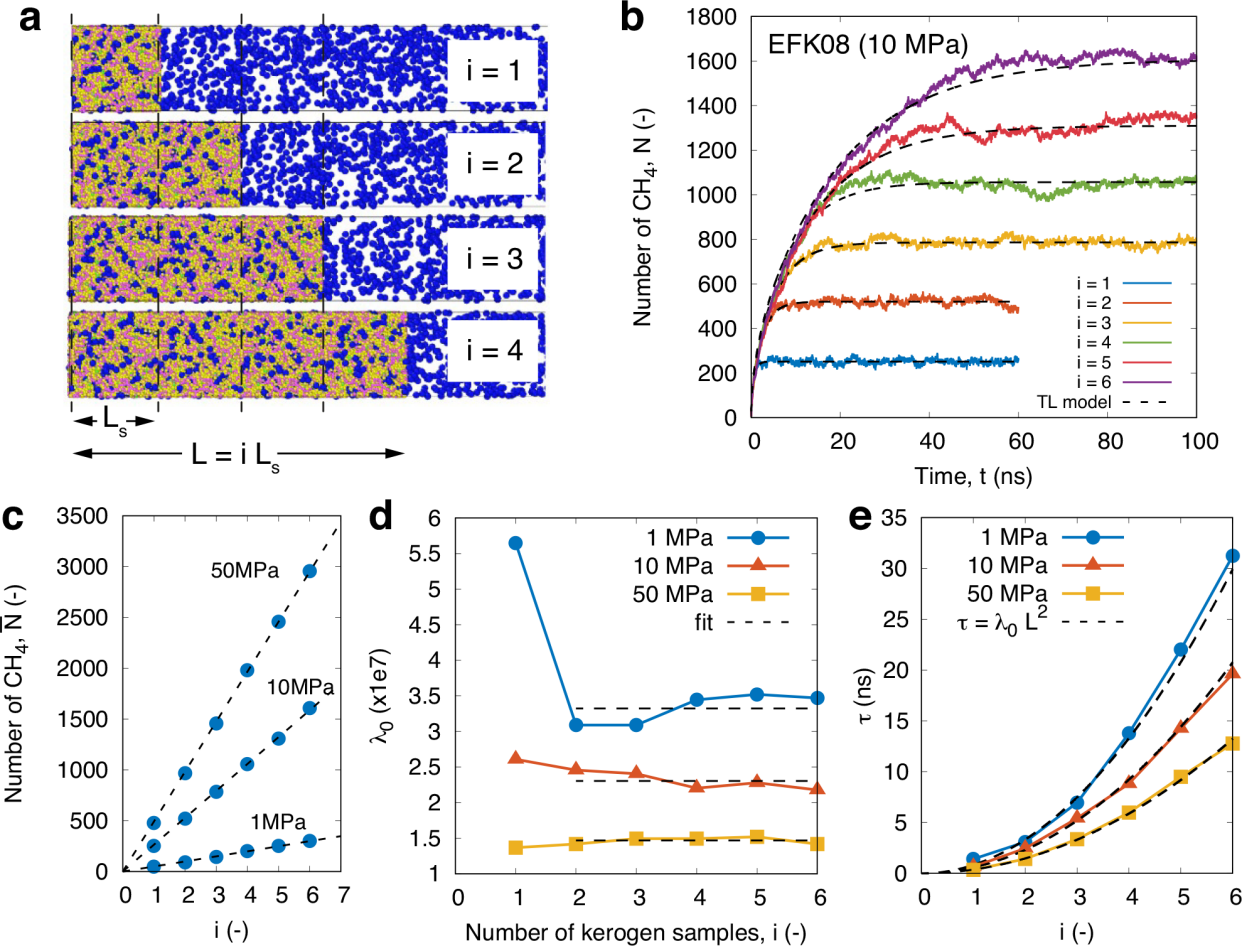
(a) Schematic of MD simulations with increasing kerogen thickness *L* = *iL*_s_, where *i* = 1–6 and *L*_s_ = 50 Å. (b) MD data for *N*(*t*) for EFK of 0.8 g/cm^3^ (EFK08) kerogen with different lengths (*L* = 50–300 Å) and *P* = 10 MPa. MD data (colored lines) are fitted using the TL model (eq 1; dashed black lines). (c) Steady-state measurements of . (d) MD data for λ_0_ = τ/*L*^2^ with horizontal fits from *i* ≥ 2. (e) The adsorption time constant versus kerogen thickness for three pressures, showing the corresponding agreement of eq 2. Values for λ_0_ are taken from fits from (d).

In this section, we verify the upscaling characteristics of our adsorption kinetics model, which is carried out by varying the thickness of the kerogen slab *L* = *iL*_s_, where *i* is the number of unit-cell kerogen replicas and *L*_s_ = 50 Å is the unit-cell size (i.e., at *i* = 1); see schematic in Figure 4a. With these being very computationally expensive MD simulations, we limit this detailed analysis to one kerogen sample only (EFK of 0.8 g/cm^3^) until *i* = 6, but checks have been made on all other samples until *i* = 3 (see results in next section). Figure 4 summarizes the key results.

Figure 4b shows that the fits using the TL model look visually good when compared with the MD results. Figure 4c verifies that steady-state adsorption is linearly proportional to *L*, and so, there are no finite-size effects in our simulations. Figure 4d shows the MD data for the kerogen/gas-dependent constant λ_0_ (eq 2). We find that the value of λ_0_ is roughly constant across all pressures for samples as small as *i* = 2. However, for the *i* = 1 sample, we observe deviations of λ_0_ above (or below) the mean horizontal line. Similar observations are seen in all the other samples. We believe this is due to the statistical noise when fitting for τ, which is resolved by increasing the length *L*. Further improvements to noise reduction could be made in the future by increasing the number of copies of the kerogen in the *y*/*z* directions, albeit at a large computational cost. Figure 4e verifies the *L*^2^ dependence scaling law in eq 2.

### Adsorption Time Scales of Different Kerogen Porosities and Maturities

**Figure 5:**
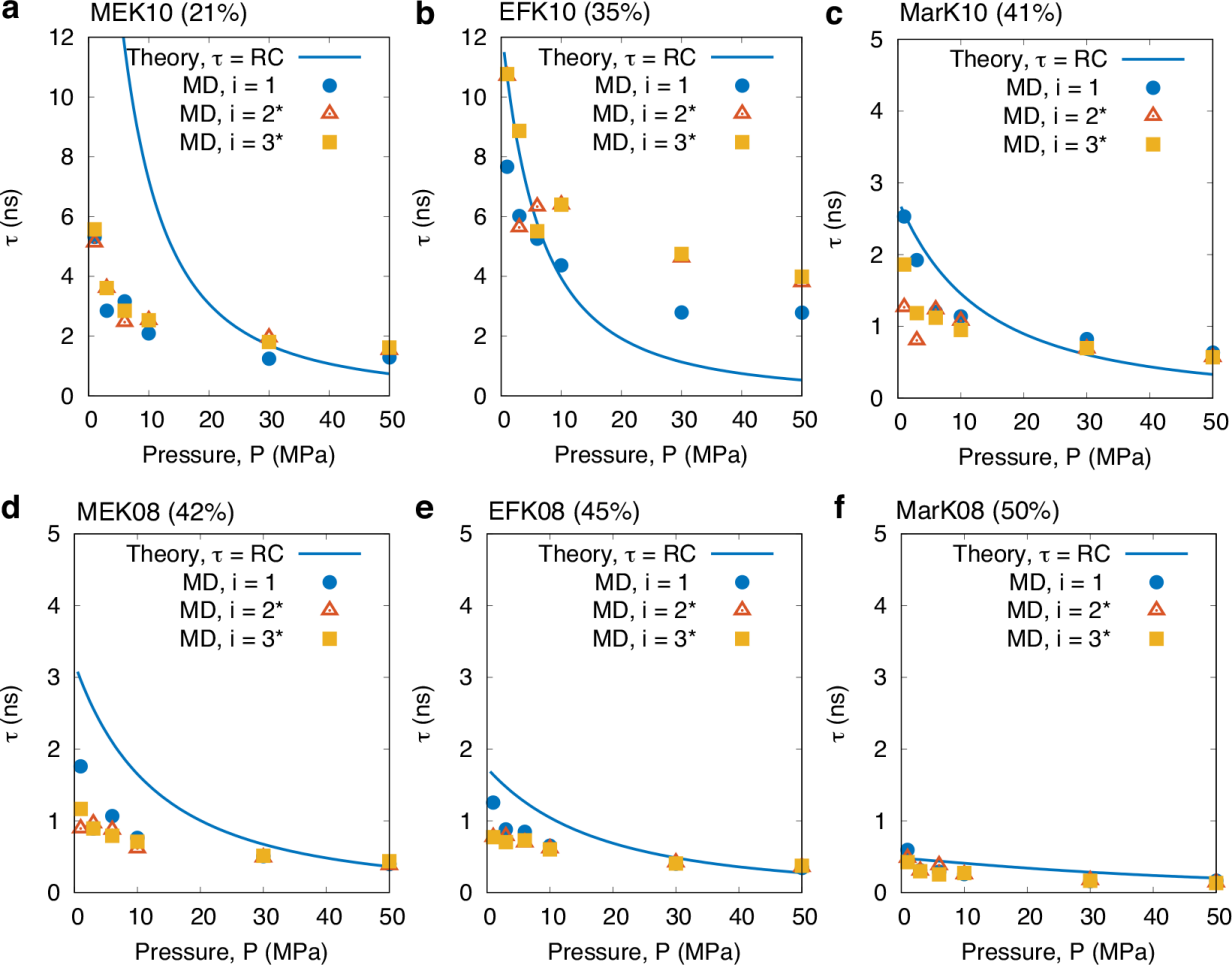
Effect of porosity (at *T* = 423 K) on the interfacial adsorption time constant for all samples in order of increasing porosity: (a) MEK10 (ϕ = 21%), (b) EFK10 (ϕ = 35%), (c) MarK10 (ϕ = 41%), (d) MEK08 (ϕ = 42%), (e) EFK08 (ϕ = 45%), and (f) MarK08 (ϕ = 50%). Symbols are our MD results, fitted using the TL model. ^★^We add data from thicker samples *i* = 2 and *i* = 3 but rescale them to *i* = 1 using the scaling law, eq 2. Solid lines are predictions from the theoretical model given in eq 5.

**Figure 6:**
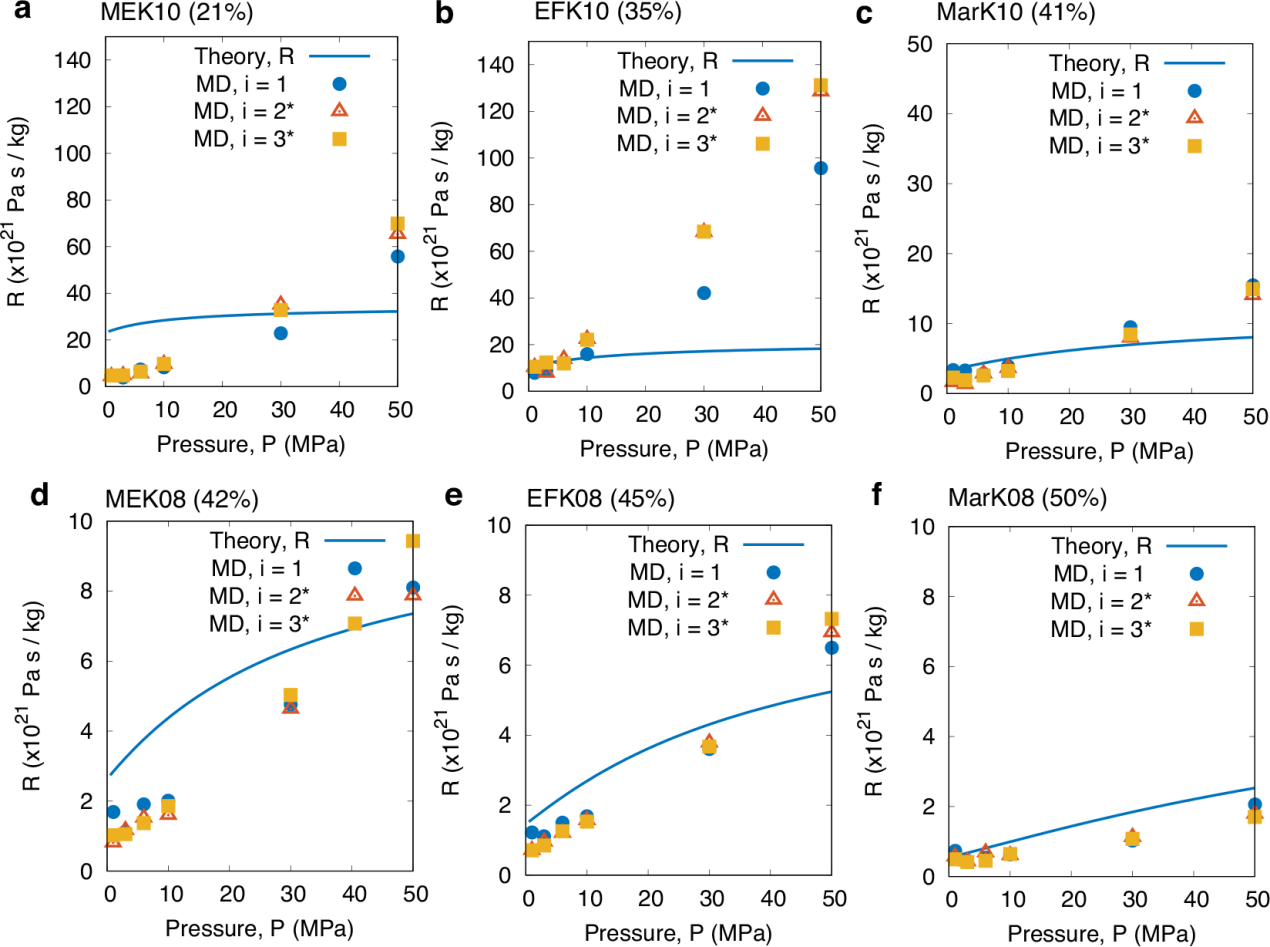
Effect of porosity (at *T* = 423 K) on the estimated total flow resistance *R* for all samples in order of increasing porosity: (a) MEK10 (ϕ = 21%), (b) EFK10 (ϕ = 35%), (c) MarK10 (ϕ = 41%), (d) MEK08 (ϕ = 42%), (e) EFK08 (ϕ = 45%), and (f) MarK08 (ϕ = 50%). Symbols are our MD results, using *R* ≈ τ/*C*, where *C* and τ are obtained from Figures 7 and 5, respectively. ^★^We add data from thicker samples *i* = 2 and *i* = 3 but rescale them to *i* = 1 using the scaling law, eq 2. Solid lines are predictions from the theoretical model given in eq 6.

**Figure 7:**
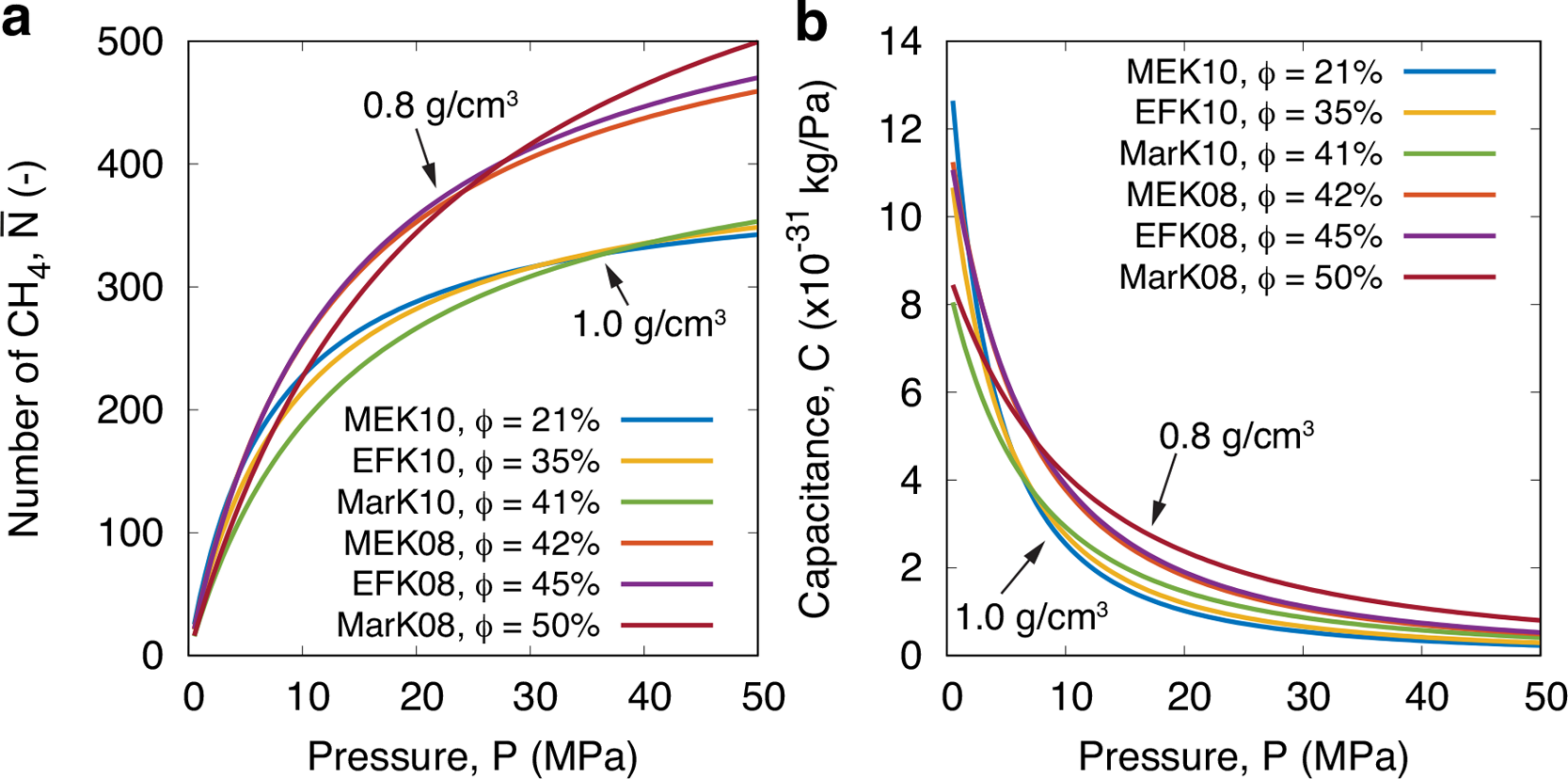
Effect of porosity (at *T* = 423 K) on (a) the steady-state number of adsorbed methane (only Langmuir fits are shown) and (b) kerogen capacitance (eq 7) for all kerogen samples considered in this work.

In this section, we show our MD results for the adsorption time constant for all kerogen samples (of varying porosity and maturity) at fixed temperature (*T* = 423 K). We include results for three kerogen thicknesses (*i* = 1, 2, 3), but those for *i* = 2 and *i* = 3 are scaled to *i* = 1, using eq 2, since it is the most fundamental unit cell considered. This also has the advantage of demonstrating that our results are size independent for *i* ≥ 2. The TL model (eq 1) is only used to obtain τ from the MD. We then compare these results with the theoretical model τ ≈ *RC*. Figures 5–7 show the key results of the full data set of kerogen samples and pressures.

Figure 5 shows the adsorption time constant τ measurements for the six kerogen samples we considered in order of increasing porosity, while Figure 6 shows the *estimated* resistances *R*. Note that we are not able to measure *R* in our MD simulations due to the prohibitive computational cost (see details in the SI). Instead, we calculate these from the relationship *R* ≈ τ/*C*, using knowledge of both τ (from the TL model fit; Figure 5) and *C* (from the Langmuir fit; Figure 7). The results for *R* are presented here for completeness, although they require more rigorous testing using independent transport simulations, in particular to verify the higher pressures. In the SI, we carry out basic checks using independent MD transport simulations for one kerogen sample and low pressures and verify that the *R*’s we are getting using this approach are close and also match well with the theoretical values of *R*. Finally, it is worth stressing that the theoretical *R*’s (solid lines in Figure 6) are independently calculated using eq 6.

The results in Figure 5 reveal that adsorption is faster with an increase in ϕ at most pressures, despite there being more molecules to adsorb. For example, comparing a 10% reduction in porosity between EFK08 and EFK10, we see that  (Figure 7a) and *C* (Figure 7b) are always larger for EFK08 at any pressure, by ∼50% or less. However, *R* for EFK10 is at least 1 order of magnitude larger than EFK08. This proves that the kerogen’s flow resistance *R* (and so the self-diffusion coefficient) is the responsible factor that drives the increase in τ when porosity decreases and the pressure remains constant, rather than from the slight drop in adsorption capacitance *C* (see Figure 7b; purple vs yellow curves). The contribution of the adsorption capacitance *C* to the decrease in τ becomes more prominent for each individual kerogen sample in the case when pressure increases, since *R* is seen to increase with *P*, while both τ and *C* decrease with *P* (see Figures 5–7). Note that *C* represents the ability of the kerogen to store more gas molecules from a small increase in pressure, which becomes saturated as *P* increases.

Although kerogen samples with low porosity have a smaller accessible volume for adsorbing methane molecules, in some cases (e.g., MEK10 and EFK10), both  and *C* start high at low pressures for low porous samples, as shown in Figure 7a,b. This occurs due to the strong adsorption capacitance of small pores at low pressure, as we find in our recent study.^44^ As the pressure increases, however, the small pores are quickly filled, and the adsorption capacitance is then dictated by the physical accessible pore volume. At higher pressures, the high porosity kerogen samples adsorb more methane molecules and this is reflected in both the Langmuir isotherms and the adsorption capacitance curves (Figure 7a,b).

Throughout our dataset, the comparison between the theoretical model for τ (solid lines in Figures 5 and 6) and our MD results are of the same order of magnitude and show similar trends with pressure and porosity, although we note there are some quantitative differences at some points. Minor checks have been made on MEK10 and EFK10 samples, where some large disagreements in *R* are observed. We find that EFK10 has a large anisotropy effect, where transport through one face of the sample is much slower than the other two, yet we can only currently use the full-dimension self-diffusion coefficient in the theoretical model for τ. No anisotropy is found in the other samples. MEK10 has very minor connectivity discrepancies, and this is not surprising since its porosity is below the minimum percolation threshold of 23%.^17^

Given the historical challenges of predicting the transport in complex nanoconfined flow networks, the theoretical model does a decent job. The discrepancies are also not unusual, given that ultratight pores, even in very simple pipe/planar geometries, are also difficult to get a perfect agreement with theoretical results for transport or self-diffusion.^30,45^

The theoretical model we choose for τ ≈ *RC* as well as the use of steady-state models for *R* and *C* to predict transient solutions are assumptions that need to be challenged in the future, even though we have calibrated each term using independent but representative MD data of the same kerogen and gas combination. For example, one suggestion would be that the values for self-diffusion taken from Obliger et al.^17^ for rigid samples, on which our *R* depends, could be carefully recalibrated for a flexible matrix. Further improvements could be made by understanding how to incorporate the effect of nonlinearities arising from *R* and *C* with pressure and taking further inspiration from electrical circuit theory to improve the theoretical model.

## Conclusions

Nonequilibrium molecular dynamics simulations are used to measure the time-varying adsorption of methane in organic tight porous rock, known as kerogen. We propose a sorption kinetics model that is able to capture the time variation of the gas inside kerogen with the MD very well. The sorption kinetics model has also allowed us to rationalize the process using the adsorption time constant τ, which we do for all pressures relevant to shale, and various samples from different geological conditions (fields, maturities, porosities). Our results show there is a clear correlation between porosity, pressure, and τ: higher pressures and higher porosities have faster adsorption time scales. We attribute this to a decrease in flow resistance *R* (higher diffusion) of methane inside the matrix, despite having a higher adsorption capacitance *C* for larger porosities. However, we also show that the adsorption capacitance is important when pressure is varied for single kerogen samples, since τ and *C* both drop with pressure, as *R* increases. This arises from the nonlinear profile of the adsorption isotherm at higher pressures. In this analysis, we have adopted an approximate theoretical model for τ, which is inspired from electrical circuitry. This model gave good qualitative agreement with our results but may need to be refined further for better predictive accuracy. The theoretical model for τ could also be used to guide future experiments.

This work now provides a route to implement new flux boundary conditions that model the time-varying sorption processes in higher scale flow models, where the TL model and the calibration of MD data presented can be used. Advanced flow models^28−31^ currently being developed to model the three-dimensional low-speed, rarefied-to-dense gas flows inside shale rock are currently missing these types of boundary models. Therefore, simulations showing the effect of time-varying adsorption/desorption processes near kerogen boundaries on the transport inside the larger meso/macropores can now be studied. This work also produces a protocol for studying the behavior of other gases or liquids in shales that are becoming important for stimulation, as well as reservoir gas storage schemes, such as for carbon dioxide sequestration or hydrogen energy storage.

## Data Availability

Sample LAMMPS files and the raw MD adsorption simulation data are available open access at https://doi.org/10.7488/ds/3805.

## Supporting Information Available

Assessment of rigid vs thermal modeling of kerogen atoms on the adsorption kinetics; derivations of the interfacial sorption kinetics model and the adsorption time constant model; validation study of the transport model in kerogen; generated MD adsorption data (PDF)
