# Supplementary material for: Interfacial Adsorption Kinetics of Methane in Microporous Kerogen
Source: Langmuir. 2023 Mar 1;39(10):3742–51. doi: 10.1021/acs.langmuir.2c03485 (PMC10018763; doi:10.1021/acs.langmuir.2c03485)
Supplement: Supplementary file 1 — la2c03485_si_001.pdf [file la2c03485_si_001.pdf]

Supporting Information: Interfacial adsorption kinetics of methane in microporous kerogen

Runxi Wang,*,† Saikat Datta,*,‡ Jun Li,*,¶ Saad F. K. Al-Afnan,*,¶ Livio Gibelli,*,‡ and Matthew K. Borg*,‡

†*Institute of New Energy and Low-carbon Technology, Sichuan University, Chengdu, 610065, China*

‡*School of Engineering, Institute of Multiscale Thermofluids, The University of Edinburgh, Edinburgh EH9 3FB, UK*

¶*Center for Integrative Petroleum Research, College of Petroleum Engineering and Geosciences, King Fahd University of Petroleum and Minerals, Dhahran 31261, Saudi Arabia*

E-mail: runxi.wang@scu.edu.cn; saikat.mech@gmail.com; lijun04@gmail.com; safnan@kfupm.edu.sa; livio.gibelli@ed.ac.uk; matthew.borg@ed.ac.uk

Contents

1 Assessment of rigid modelling of kerogen atoms on the adsorption kinetics S-2

2 Interfacial sorption kinetics model: derivation S-5

3 Theoretical model for τ S-6

3.1 *Capacitance* S-7

3.2 *Resistance* S-7

4 Validation of transport model R **S-9**

5 MD adsorption data **S-11**

References **S-13**

1 Assessment of rigid modelling of kerogen atoms on the adsorption kinetics

An important finding in our MD methodology is the observed differences in the adsorption timescales between rigid (i.e. adiabatic) and flexible (i.e. thermal) descriptions of the kerogen. Figures S1a-d show $N(t)$ in both rigid and flexible EFK 0.8 g/cm^3 kerogen samples after different step pressures (1, 10, 30, 50 MPa) are applied to the reservoir. Notably, while the same steady-state $\overline{N}$ is achieved, we find that the sorption processes in the rigid kerogen are slower than the flexible kerogen, as seen by careful inspection of Figure S1a-d and more clearly in Figure S1e. Recent studies have found differences in self-diffusion between flexible and rigid kerogen models,[S1,S2] which originate from a combination of swelling and tight flexible constrictions that open/close within the atomic matrix. In our study, where methane adsorbs at an interface between a reservoir and the kerogen, we find that the type of kerogen model plays an important role that differs from these mechanisms, which we explain below. It is worth highlighting that our kerogen model does not implement swelling. In this section, we also include the equivalent desorption results, to show that the same effect is also observed, although the main focus of the paper is on adsorption.

The observed differences are explained from the thermodynamics of the adsorption/desorption processes. In all our simulations, the pressure and temperature of the reservoir remain constant with time. However, the pressure, temperature and number of methane molecules

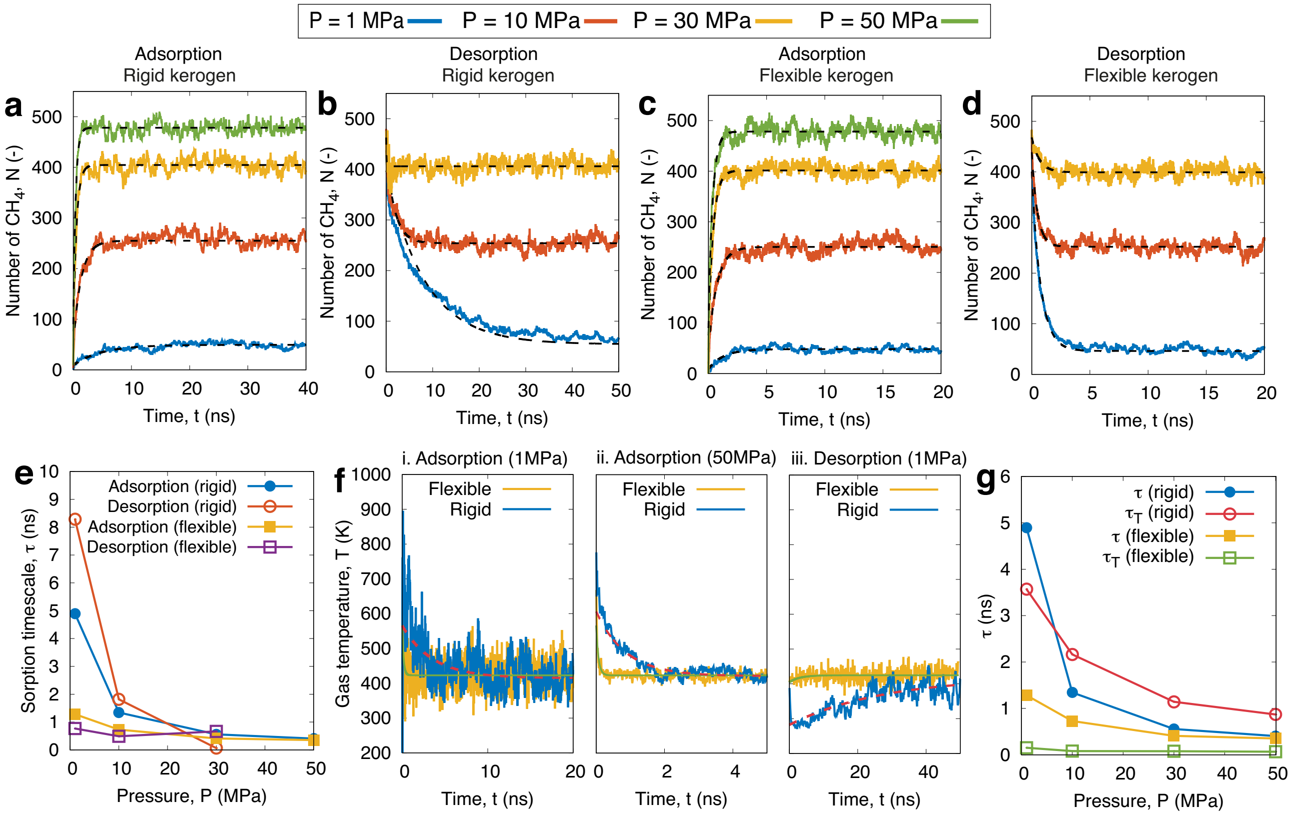

Figure S1: Time evolution of the number of methane molecules inside the EFK 0.8 g/cm^3 kerogen for selected pressures for (a) adsorption of methane inside a rigid-modelled kerogen, and (b) equivalently the desorption stages, (c) adsorption of methane in a flexible kerogen set at temperature $T = 423$ K, and (d) equivalently the desorption stages. The black dashed line is the fitting result of equation (4). (e) Comparison of the time constants of methane adsorption and desorption processes. (f) Time evolution of the temperature of methane molecules inside the kerogen for (i.) adsorption ($0 \rightarrow 1$ MPa), (ii.) ($0 \rightarrow 50$ MPa) and (iii.) desorption (50 MPa $\rightarrow$ 1 MPa). Fitted green and red lines use same form as equation (4). (g) Comparison of the thermal time constant and adsorption time constant.

inside the kerogen changes during adsorption/desorption. While pressure is more challenging to compute in confined geometries, we show measurements of temperature in Figures S1f(i.–iii.) for adsorption (0 $\rightarrow$ 1 MPa and 0 $\rightarrow$ 50 MPa) and desorption (50 $\rightarrow$ 1 MPa). Notably, from the Gay-Lussac's law, $P_1/T_1 = P_2/T_2$ applied only to the kerogen (where volume is constant), the local gas temperature inside the kerogen T_2 is expected to increase during the initial time period of adsorption (while P_2 increases), and decrease during desorption (while P_2 decreases), which confirms our results.

By visual inspection of Figures S1f(i.–iii.), the temperature of methane adsorbed in a rigid sample takes longer to settle back to the target reservoir temperature. To demonstrate this quantitatively, we define a thermal time constant τ_T, measured using the same form of equation (4), with temperature T replacing N. The results in Figure S1g show that for flexible kerogen, $\tau_T \ll \tau$. This means that methane collisions with the thermalised kerogen atoms rapidly accommodate to its temperature, helping the system reach a thermal equilibrium faster and does not affect the adsorption timescale τ. However, for the rigid kerogen, $\tau_T \approx \tau$, which implies that the sorption processes are limited by the artificially slow thermal timescales. Note that in the rigid set-up, the Nosé-Hoover thermostat is applied globally to all methane molecules (reservoir included) as would be required in a canonical ensemble. Therefore, because of the reservoir's size, any local increase/decrease in temperature inside a kerogen matrix modelled adiabatically, would not dissipate quickly using this approach. Instead, thermalisation of the internal fluid relies on the transport of further gas molecules coming in from the reservoir at a temperature T, as well as the gas-gas collisions. This also explains why the desorption timescales in the rigid case are longer, since more molecules are leaving the kerogen than those coming in, which are responsible for the thermalisation.

2 Interfacial sorption kinetics model: derivation

Recent work[S3,S4] have shown that the steady transport process of hydrocarbons and carbon dioxide within nanopores is diffusion-based. Therefore, the model that is closest to describing the time-varying sorption processes for our MD set-up, shown in Figure S2a, is derived by solving the one-dimensional diffusion equation in a *single straight nanopore* (see Fig. S2b):

$$\frac{\partial \rho(x,t)}{\partial t} = D_{\text{eff}} \frac{\partial^2 \rho(x,t)}{\partial x^2}, \tag{1}$$

where x is the direction of adsorption, ρ is methane density inside the nanopore averarged over the cross-section and D_{eff} is the effective diffusion coefficient that describes the non-equilibrium sorption process. The number of adsorbed molecules inside the nanopore per unit time, $N(t)$, is given by:

$$N(t) = \int_0^L \rho(x,t)dx. \tag{2}$$

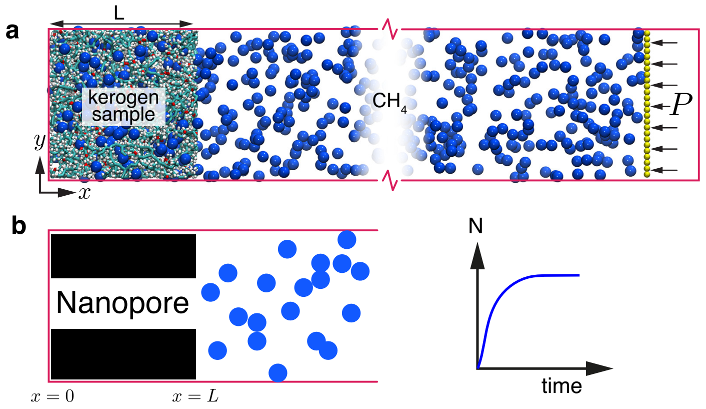

Figure S2: (a) MD set-up used in this work, (b) model of simple nanopore used to derive a diffusion equation for $N(t)$.

For an adsorption process that best replicates our set-up, the nanopore would be unloaded at the beginning of the simulation, so $\rho(x,0) = 0$. At the point the nanopore is momentarily opened, the boundary condition at the reservoir–nanopore interface is $\rho(x = L, t > 0) = \overline{\rho}$, where $\overline{\rho}$ is the steady and constant reservoir density. Since no methane molecules escape

through the closed end of the nanopore ($x = 0$; see Figure S2b), the Neumann boundary condition can be applied $\partial\rho/\partial x|_{x=0} = 0$. From the solution of equation (1), considering these initial and boundary conditions, and extending it to a general form for both adsorption and desorption, the time-varying sorption of gas inside the nanopore (i.e. equation (2)) can be expressed as:[S5]

$$N(t) = \Delta N \left[\frac{2}{\pi^2} \sum_{n=0}^{\infty} \frac{\exp\left(-\pi^2 (n+\frac{1}{2})^2 (\frac{2l}{L})^2 \frac{t}{\tau_0}\right)}{(n+\frac{1}{2})^2} \right] + \overline{N}, \tag{3}$$

where $\Delta N = (N_0 - \overline{N})$ is the change in the number of adsorbed gas molecules at time $t = 0$ (given by N_0) and the steady-state (given by $\overline{N}$, $t \to \infty$), L is the length of the nanopore and l is the nanopore volume divided by its surface area. Note that τ_0 here is the intrinsic time constant ($\tau_0 = 4l^2/D_{\text{eff}}$),[S5–S8] which represents the intrinsic material properties of the nanopore–methane combination, and is dependent on reservoir pressure and temperature.

Given some terms inside equation (3) are too challenging to determine for a complex porous medium, instead of the single straight nanopore they were derived for, we group the term $\lambda_0 = \tau_0/(l\pi)^2$, such that the final equation describing $N(t)$ is given by:

$$N(t) = \Delta N \left[\frac{2}{\pi^2} \sum_{n=0}^{\infty} \frac{\exp\left(-(n+\frac{1}{2})^2 \frac{4t}{\lambda_0 L^2}\right)}{(n+\frac{1}{2})^2} \right] + \overline{N}, \tag{4}$$

where the term $\lambda_0 L^2$ is the adsorption time constant τ.

3 Theoretical model for τ

As described in the manuscript, the theoretical model for the adsorption time constant is taken to be:

$$\tau \approx RC. \tag{5}$$

3.1 *Capacitance*

The capactiance term C in equation (5) can be defined according to Langmuir[S9] through the adsorption isotherm, which describes the amount of fluid stored inside a porous structure as a function of reservoir pressure, at fixed temperature:

$$M(P) = M_\infty \frac{K_0 P}{1 + K_0 P}, \tag{6}$$

where $M = mN$ is the mass of adsorbed methane under a certain pressure, and M_∞ is the saturated mass of adsorbed methane molecules. K_0 is the equilibrium constant (in units of inverse pressure) and P is the pressure. Equation (6) can be calibrated from molecular simulations,[S10–S12] experiments,[S13,S14] or semi-empirically.[S15] Our definition of C is based on the differential of the Langmuir isotherm:

$$C = \frac{dM}{dP} = M_\infty \frac{K_0}{(1 + K_0 P)^2}. \tag{7}$$

The form of this expression is a monotonic decrease of C with pressure.

3.2 *Resistance*

The transport resistance term R can be defined by considering recent studies in which diffusion and adsorption are found to play a crucial role in the transport mechanics inside tight porous media,[S16] including kerogen.[S3,S10] For example, Falk et al.[S3] describes a scaling law for hydrocarbons relating the permeance K with adsorption, and self-diffusion D_s through

$$K = \frac{D_s}{k_B T} \frac{V}{N}, \tag{8}$$

where k_B is the Boltzmann constant, V is the kerogen sample box volume and N are the number of adsorbed molecules, and is related in our work with flow resistance using:

$$R = \frac{L^2}{NmK} = \frac{k_B T L^2}{m D_s V}. \tag{9}$$

The self-diffusion term D_s can be obtained using equilibrium MD simulations within the free-volume framework:[S3,S10]

$$D_s = \frac{k_B T}{\xi_0} \exp\left(-\frac{\alpha\beta\Gamma}{1-\beta\Gamma}\right), \tag{10}$$

where ξ_0 is the friction coefficient between the fluid molecules and the matrix, $\Gamma = M/M_\infty$ is the loading, α is a coefficient which accounts for the overlap of the free volume for each molecule and β is the molecular packing efficiency. Obliger et al.[S10] demonstrated a universal behaviour for the parameters ξ_0, α and β as a function of porosity ϕ for a number of hydrocarbons with varying chain length and proposed a set of empirical equations. Here we use these formulas in the following modified format:

$$\alpha = a_0(\phi - \phi_c), \tag{11}$$

$$\xi_0 = k_B T \exp\left(-(a_0\phi + a_1)\right), \tag{12}$$

$$\beta = \beta_s \phi, \tag{13}$$

with fitting coefficients $a_0 = 0.197$ and $a_1 = -26.2$. In these formulas, $\phi_c = 23\%$ is the percolation threshold porosity, $\beta_s \approx 0.64/100$ is the maximum packing efficiency, while ϕ is the porosity (defined in percentage).[S10]

4 Validation of transport model R

The self-diffusion measurements inside kerogen, as detailed in Obliger et al.,[S10] did not contain a validation step to check that the data produces the correct bulk permeance in the same kerogen. Measuring transport using non-equilibrium MD is very computationally expensive due to the low permeability, and low pressure gradients that are required. We find that on closer inspection of the calibration study presented in Obliger et al.[S10] we had to make changes to the fitting equations, for α and ξ_0 (as presented earlier in equations (11) and (12)). We carry out simple checks here of the re-calibrated free-volume model in comparison with the transport case.

We use two independent set-ups for EFK 0.8 g/cm^3. The original set-up for time-varying adsorption as presented in the manuscript (or see Fig. S2). This allows us to determine the capacitance (see Fig. S3a), and the time-constant (see Fig. S3b). We then use a Fick's MD set-up to measure transport through the same kerogen (see new set-up inset Fig. S3c). A reservoir is placed on either side of the kerogen. The piston in each reservoir are set at a different pressure P_1 (left reservoir) and P_2 (right reservoir) such to apply a pressure drop $\Delta P = P_1 - P_2 \sim 3$ MPa across the kerogen sample. For each case we measure total flow resistance $R = \Delta P/\dot{m}$, where $\dot{m}$ is the measured mass flow rate. We vary the mean pressure $\overline{P} = (P_1 + P_2)/2$ and the length of the kerogen sample. This allows us to calculate the bulk resistance as shown in Figure S3c for a few mean pressures. Note that due to the extremely low permeability at higher pressures, the thermal noise is far too high to obtain enough statistics of mass flow rate, and so we avoid these large pressures. The same can be said for lower porosity samples, where the equivalent lower porous sample EFK10 has a one order of magnitude increase in flow resistance (see manuscript).

As shown in Figure S3b,d, the flow resistance and the adsorption time constant are close between our independent MD simulation results for transport and adsorption, as well as the theoretical models for R and $\tau \approx RC$.

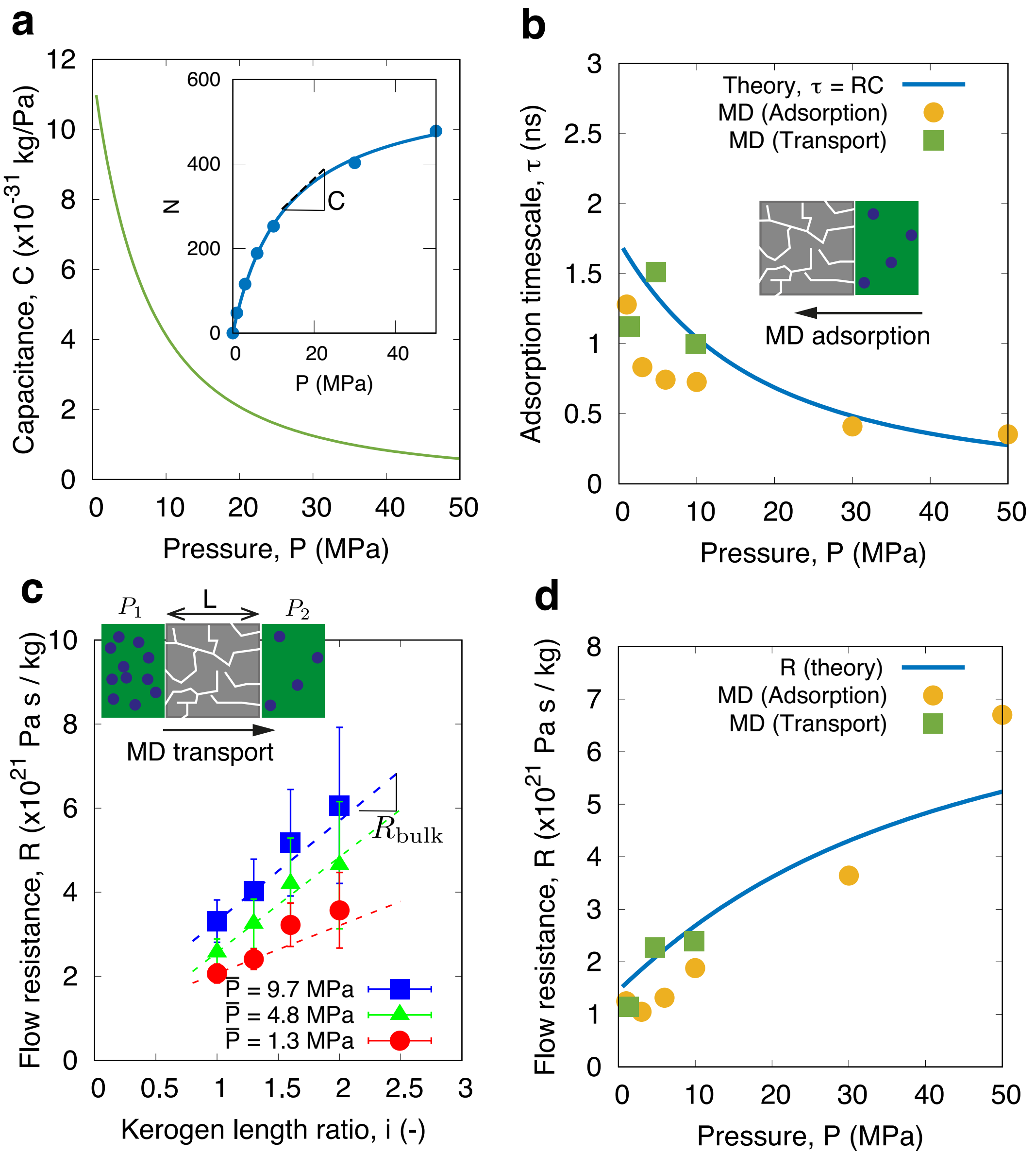

Figure S3: Validation of transport model for the EFK 0.8 g/cm^3 kerogen sample at $T = 423$ K. (a) Adsorption capacitance C (inset: adsorption isotherm). (b) Adsorption timescales measured from MD shown using yellow circle symbols (inset: schematic of MD set-up). (c) Flow resistances of methane measured from transport MD simulations of different kerogen lengths $L = iL_s$ (i is a real number and $L_s = 50$ Å is the original kerogen length) and at different mean reservoir pressures $\overline{P} = (P_1 + P_2)/2$ (inset: schematic of MD set-up); dashed lines are fits through MD data. (d) Comparison of flow resistances R as a function of pressure between our transport MD simulations (green squares), and R calculated using our re-calibration of the diffusion expression (blue solid line; equations (9)-(13)). We add estimates of R from the adsorption MD simulations using $R \approx \tau/C$ (see main paper). Similarly we add τ predictions of the transport MD case to figure (b) using $\tau \approx RC$ with R obtained from the transport MD results and C taken similarly from figure (a).

5 MD adsorption data

Tables S1-S6 give the summary of all the adsorption MD data presented in the manuscript, while Figure S4 shows the Langmuir fits with the steady-state adsorption results.

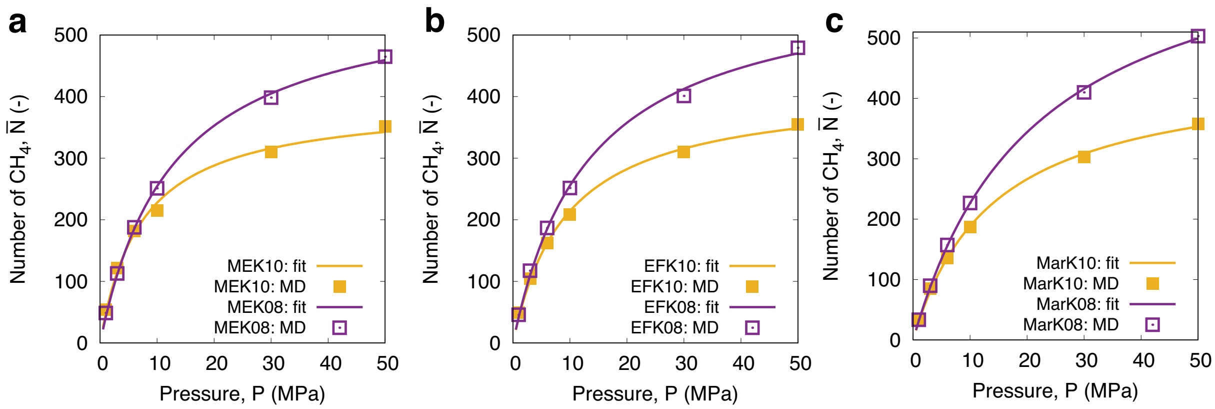

Figure S4: Results of steady adsorption from MD and Langmuir fits for (a) MEK10, MEK08, (b) EFK10, EFK08, and (c) MarK10, MarK08 kerogen samples.

Table S1: MD data from adsorption simulations for MEK08

P (MPa)	$\overline{N}$ (-)	τ (ns)	C (kg/Pa)	R (Pa s/kg)
1	50	1.17	11.39×10^{-31}	1.02×10^{21}
3	127	0.89	8.44×10^{-31}	1.06×10^{21}
6	200	0.79	5.78×10^{-31}	1.37×10^{21}
10	265	0.71	3.82×10^{-31}	1.86×10^{21}
30	407	0.51	1.02×10^{-31}	5.03×10^{21}
50	478	0.44	0.46×10^{-31}	9.43×10^{21}

Table S2: MD data from adsorption simulations for MEK10

P (MPa)	$\overline{N}$ (-)	τ (ns)	C (kg/Pa)	R (Pa s/kg)
1	62	5.57	11.69×10^{-31}	4.77×10^{21}
3	126	3.61	7.49×10^{-31}	4.82×10^{21}
6	181	2.85	4.44×10^{-31}	6.42×10^{21}
10	230	2.53	2.60×10^{-31}	9.74×10^{21}
30	326	1.80	0.55×10^{-31}	32.83×10^{21}
50	356	1.62	0.23×10^{-31}	69.94×10^{21}

Table S3: MD data from adsorption simulations for EFK08

P (MPa)	$\overline{N}$ (-)	τ (ns)	C (kg/Pa)	R (Pa s/kg)
1	48	0.77	10.90×10^{-31}	0.71×10^{21}
3	122	0.70	8.27×10^{-31}	0.85×10^{21}
6	194	0.73	5.81×10^{-31}	1.25×10^{21}
10	262	0.60	3.93×10^{-31}	1.53×10^{21}
30	412	0.41	1.10×10^{-31}	3.67×10^{21}
50	485	0.37	0.51×10^{-31}	7.32×10^{21}

Table S4: MD data from adsorption simulations for EFK10

P (MPa)	$\overline{N}$ (-)	τ (ns)	C (kg/Pa)	R (Pa s/kg)
1	54	10.77	10.28×10^{-31}	10.47×10^{21}
3	114	8.87	7.18×10^{-31}	12.35×10^{21}
6	162	5.51	4.62×10^{-31}	11.92×10^{21}
10	229	6.40	2.90×10^{-31}	22.09×10^{21}
30	329	4.75	0.69×10^{-31}	68.45×10^{21}
50	373	3.99	0.30×10^{-31}	131.19×10^{21}

Table S5: MD data from adsorption simulations for MarK08

P (MPa)	$\overline{N}$ (-)	τ (ns)	C (kg/Pa)	R (Pa s/kg)
1	36	0.42	8.62×10^{-31}	0.49×10^{21}
3	96	0.30	7.19×10^{-31}	0.41×10^{21}
6	165	0.25	5.63×10^{-31}	0.45×10^{21}
10	239	0.27	4.23×10^{-31}	0.64×10^{21}
30	423	0.16	1.53×10^{-31}	1.08×10^{21}
50	515	0.13	0.78×10^{-31}	1.70×10^{21}

Table S6: MD data from adsorption simulations for MarK10

P (MPa)	$\overline{N}$ (-)	τ (ns)	C (kg/Pa)	R (Pa s/kg)
1	37	1.86	8.13×10^{-31}	2.29×10^{21}
3	92	1.18	6.17×10^{-31}	1.92×10^{21}
6	147	1.12	4.34×10^{-31}	2.58×10^{21}
10	194	0.95	2.94×10^{-31}	3.24×10^{21}
30	306	0.70	0.82×10^{-31}	8.45×10^{21}
50	365	0.57	0.38×10^{-31}	14.93×10^{21}

References

(S1) Wu, T.; Firoozabadi, A. Effect of Microstructural Flexibility on Methane Flow in Kerogen Matrix by Molecular Dynamics Simulations. The Journal of Physical Chemistry C **2019**, 123, 10874–10880.

(S2) Obliger, A.; Valdenaire, P.-L.; Ulm, F.-J.; Pellenq, R. J.-M.; Leyssale, J.-M. Methane diffusion in a flexible kerogen matrix. The Journal of Physical Chemistry B **2019**, 123, 5635–5640.

(S3) Falk, K.; Coasne, B.; Pellenq, R.; Ulm, F.-J.; Bocquet, L. Subcontinuum mass transport of condensed hydrocarbons in nanoporous media. Nature communications **2015**, 6, 1–7.

(S4) Zhang, H.; Wang, S.; Yin, X.; Qiao, R., et al. Soaking in CO2 huff-n-puff: A single-nanopore scale study. Fuel **2022**, 308, 122026.

(S5) Wang, R.; Bi, S.; Guo, Z.; Feng, G. Molecular insight into replacement dynamics of CO2 enhanced oil recovery in nanopores. Chemical Engineering Journal **2022**, 440, 135796.

(S6) Kornyshev, A. A.; Twidale, R. M.; Kolomeisky, A. B. Current-generating double-layer shoe with a porous sole: ion transport matters. The Journal of Physical Chemistry C **2017**, 121, 7584–7595.

(S7) Bi, S.; Banda, H.; Chen, M.; Niu, L.; Chen, M.; Wu, T.; Wang, J.; Wang, R.; Feng, J.; Chen, T., et al. Molecular understanding of charge storage and charging dynamics in supercapacitors with MOF electrodes and ionic liquid electrolytes. Nature Materials **2020**, 19, 552–558.

(S8) Péan, C.; Merlet, C.; Rotenberg, B.; Madden, P. A.; Taberna, P.-L.; Daffos, B.;

Salanne, M.; Simon, P. On the Dynamics of Charging in Nanoporous Carbon-Based Supercapacitors. ACS Nano **2014**, 8, 1576–1583, PMID: 24417256.

(S9) Langmuir, I. The adsorption of gases on plane surfaces of glass, mica and platinum. Journal of the American Chemical Society **1918**, 40, 1361–1403.

(S10) Obliger, A.; Ulm, F. J.; Pellenq, R. Impact of Nanoporosity on Hydrocarbon Transport in Shales' Organic Matter. Nano Letters **2018**, 18, 832–837.

(S11) Zhao, T.; Li, X.; Zhao, H.; Li, M. Molecular simulation of adsorption and thermodynamic properties on type II kerogen: Influence of maturity and moisture content. Fuel **2017**, 190, 198–207.

(S12) Sui, H.; Zhang, F.; Wang, Z.; Wang, D.; Wang, Y. Effect of kerogen maturity, water content for carbon dioxide, methane, and their mixture adsorption and diffusion in kerogen: a computational investigation. Langmuir **2020**, 36, 9756–9769.

(S13) Bousige, C.; Ghimbeu, C. M.; Vix-Guterl, C.; Pomerantz, A. E.; Suleimenova, A.; Vaughan, G.; Garbarino, G.; Feygenson, M.; Wildgruber, C.; Ulm, F.-J.; Pellenq, R. J. M.; Coasne, B. Realistic molecular model of kerogen's nanostructure. Nature Materials **2016**, 15, 576–582.

(S14) Li, J.; Zhou, S.; Gaus, G.; Li, Y.; Ma, Y.; Chen, K.; Zhang, Y. Characterization of methane adsorption on shale and isolated kerogen from the Sichuan Basin under pressure up to 60 MPa: Experimental results and geological implications. International Journal of Coal Geology **2018**, 189, 83–93.

(S15) Wang, R.; Li, J.; Gibelli, L.; Guo, Z.; Borg, M. K. Sub-nanometre pore adsorption of methane in kerogen. Chemical Engineering Journal **2021**, 426, 130984.

(S16) Ziarani, A. S.; Aguilera, R. Knudsen's permeability correction for tight porous media. Transport in porous media **2012**, 91, 239–260.
